# The Role of Nonapoptotic Programmed Cell Death — Ferroptosis, Necroptosis, and Pyroptosis — in Pancreatic Ductal Adenocarcinoma Treatment

**DOI:** 10.3389/fonc.2022.872883

**Published:** 2022-05-19

**Authors:** Sheng-Kai Hsu, Yi-Hsuan Chu, Wun-Jyun Syue, Hugo You-Hsien Lin, Wen-Tsan Chang, Jeff Yi-Fu Chen, Chang-Yi Wu, Chia-Hung Yen, Kai-Chun Cheng, Chien-Chih Chiu

**Affiliations:** ^1^ Department of Biotechnology, Kaohsiung Medical University, Kaohsiung, Taiwan; ^2^ Department of Medicine, Kaohsiung Medical University, Kaohsiung, Taiwan; ^3^ Division of Nephrology, Department of Internal Medicine, Kaohsiung Medical University Hospital, Kaohsiung Medical University, Kaohsiung, Taiwan; ^4^ Division of General and Digestive Surgery, Department of Surgery, Kaohsiung Medical University Hospital, Kaohsiung, Taiwan; ^5^ Department of Biological Sciences, National Sun Yat-sen University, Kaohsiung, Taiwan; ^6^ The Graduate Institute of Natural Products, Kaohsiung Medical University, Kaohsiung, Taiwan; ^7^ Department of Ophthalmology, Kaohsiung Municipal Siaogang Hospital, Kaohsiung, Taiwan; ^8^ Department of Ophthalmology, Kaohsiung Medical University Hospital, Kaohsiung, Taiwan; ^9^ Department of Ophthalmology, School of Medicine, College of Medicine, Kaohsiung Medical University, Kaohsiung, Taiwan; ^10^ Center for Cancer Research, Kaohsiung Medical University, Kaohsiung, Taiwan; ^11^ Department of Medical Research, Kaohsiung Medical University Hospital, Kaohsiung, Taiwan; ^12^ The Graduate Institute of Medicine, Kaohsiung Medical University, Kaohsiung, Taiwan

**Keywords:** pancreatic ductal adenocarcinoma (PDAC), antiapoptotic effect, chemoresistance, ferroptosis, necroptosis, pyroptosis

## Abstract

Pancreatic ductal adenocarcinoma (PDAC) is the most lethal cancer, with a dismal 5-year survival rate of less than 10%. It is estimated that approximately 80% of pancreatic ductal carcinoma (PDAC) patients are diagnosed at an advanced or metastatic stage. Hence, most patients are not appropriate candidates for surgical resection and therefore require systemic chemotherapy. However, it has been reported that most patients develop chemoresistance within several months, partly because of antiapoptotic mechanisms. Hence, inducing alternative programmed cell death (PCD), including ferroptosis, necroptosis or pyroptosis, seems to be a promising strategy to overcome antiapoptosis-mediated chemoresistance. In this review, we shed light on the molecular mechanisms of ferroptosis, necroptosis and pyroptosis and suggest several potential strategies (e.g., compounds and nanoparticles [NPs]) that are capable of triggering nonapoptotic PCD to suppress PDAC progression. In conclusion, these strategies might serve as adjuvants in combination with clinical first-line chemotherapies to improve patient survival rates.

## Introduction

Pancreatic neoplasm is a lethal and aggressive cancer with a dismal 5-year survival rate of only 10% (1). Pancreatic ductal adenocarcinoma (PDAC) is the most prevalent pancreatic cancer, constituting 90% of all pancreatic malignancies (2). The poor prognosis in PDAC is mainly attributed to difficulties with early detection and insurmountable treatment resistance (3, 4). It is estimated that approximately 80% of patients with PDAC are diagnosed at an advanced or metastatic stage. Since fewer than 20% of patients are appropriate candidates for surgical resection, most patients require systemic chemotherapy (5). Gemcitabine (GEM) combined with nanoalbumin-bound paclitaxel (nab-PTX) or FOLFRINOX (consisting of leucovorin, 5-FU, irinotecan and oxaliplatin) is recognized as the first-line treatment for advanced PDAC (6). Nevertheless, most patients have been reported to be refractory to chemotherapies, and antiapoptotic effects are among the causes of chemoresistance (7). Importantly, it has been estimated that 90% of PDAC patients harbor KRAS mutations; mutant KRAS exerts anti-apoptotic effects through downregulation of pro-apoptotic proteins (e.g., BIM and Bax) and upregulation of anti-apoptotic proteins (e.g., Bcl-2) (8–10). Furthermore, Mikamori et al. demonstrated that prolonged GEM exposure induces overexpression of miR-155, enhancing anti-apoptotic effects (11). Intriguingly, the tumor microenvironment (TME) also contributes to chemoresistance. Recent research has elucidated that activated pancreatic stellate cells (PSCs), constituting a cell population in stroma, drive upregulated ATP-binding cassette transporter (ABC transporter) and Bcl-2 protein action against apoptosis in PDAC (12). Based on this, bypassing the apoptosis signaling pathway to induce alternative programmed cell death (PCD) seems to be a feasible strategy. Notably, a large body of evidence has revealed that inducing nonapoptotic PCD might alleviate and possibly overcome drug resistance mediated by defective apoptosis (13). In addition, nonapoptotic PCD is immunogenic, indicating its potential to modulate TME immunity (14). In summary, administration of agents that induce nonapoptotic PCD (e.g., ferroptosis, necroptosis or pyroptosis) seems to be a promising strategy to subvert treatment resistance. Hence, in this review, we shed light on the antitumor effect of nonapoptotic PCD in PDAC.

## The Role of Ferroptosis in PDAC Treatment

### Molecular Mechanism of Ferroptosis

Ferroptosis is a type of iron-dependent nonapoptotic PCD characterized by excessive iron accumulation, increased oxidative stress, and enhanced lipid peroxidation (15). Additionally, it is also a caspase-independent and immunogenic cell death (16, 17). Systemic Xc^-^, consisting of solute carrier family 3 member 2 (SLC3A2) and solute carrier family 7 member 11 (SLC7A11), serves as a cystine-glutamate antiporter. Systemic Xc^-^ involves glutamate export and cystine uptake, which facilitate glutathione (GSH) synthesis to curb reactive oxidative species (ROS) generation (18, 19). Glutathione peroxidase 4 (GPX4) is an antioxidant enzyme capable of converting GSH into glutathione disulfide (GSSG), which plays a central role in suppressing ROS generation and lipid peroxidation (15). Regarding iron homeostasis, ferritin is an intracellular iron storage protein comprising ferritin heavy polypeptide 1 (FTH1) and ferritin light polypeptide 1 (FTL1) (20). Although iron is an important trace element in physiological homoeostasis, iron accumulation induces toxicity in cells *via* ROS production through the Fenton reaction (21). Previously reported evidence has indicated that ferroptosis can be activated by autophagic degradation of ferritin to liberate iron, a process termed ferritinophagy. In addition, ferritinophagy is primarily mediated by nuclear receptor coactivator 4 (NCOA4) (**Table 1**) (22).

**Table 1:** The biological features of ferroptosis, necroptosis and pyroptosis.

	Immunogenic	Caspase-dependent	Pore formation	Pore-forming executor	Key regulators	Reference
Ferroptosis	Yes	Independent	Yes (Nanopores)	N/A	Systemic Xc^-^ GPX4 NCOA4	(15–17, 22)
Necroptosis	Yes	Independent	Yes	MLKL	RIPK1 RIPK3 MLKL	(23, 24)
Pyroptosis	Yes	Dependent	Yes	Gasdermin	**Canonical**: NLRP3 inflammasome, caspase-1, GSDMD	(25, 26)
					**Non-canonical**: caspase-4/-5/-11/-3, GSDME	

### Strategies to suppress PDAC Growth *via* Ferroptosis

**Figure 1:**
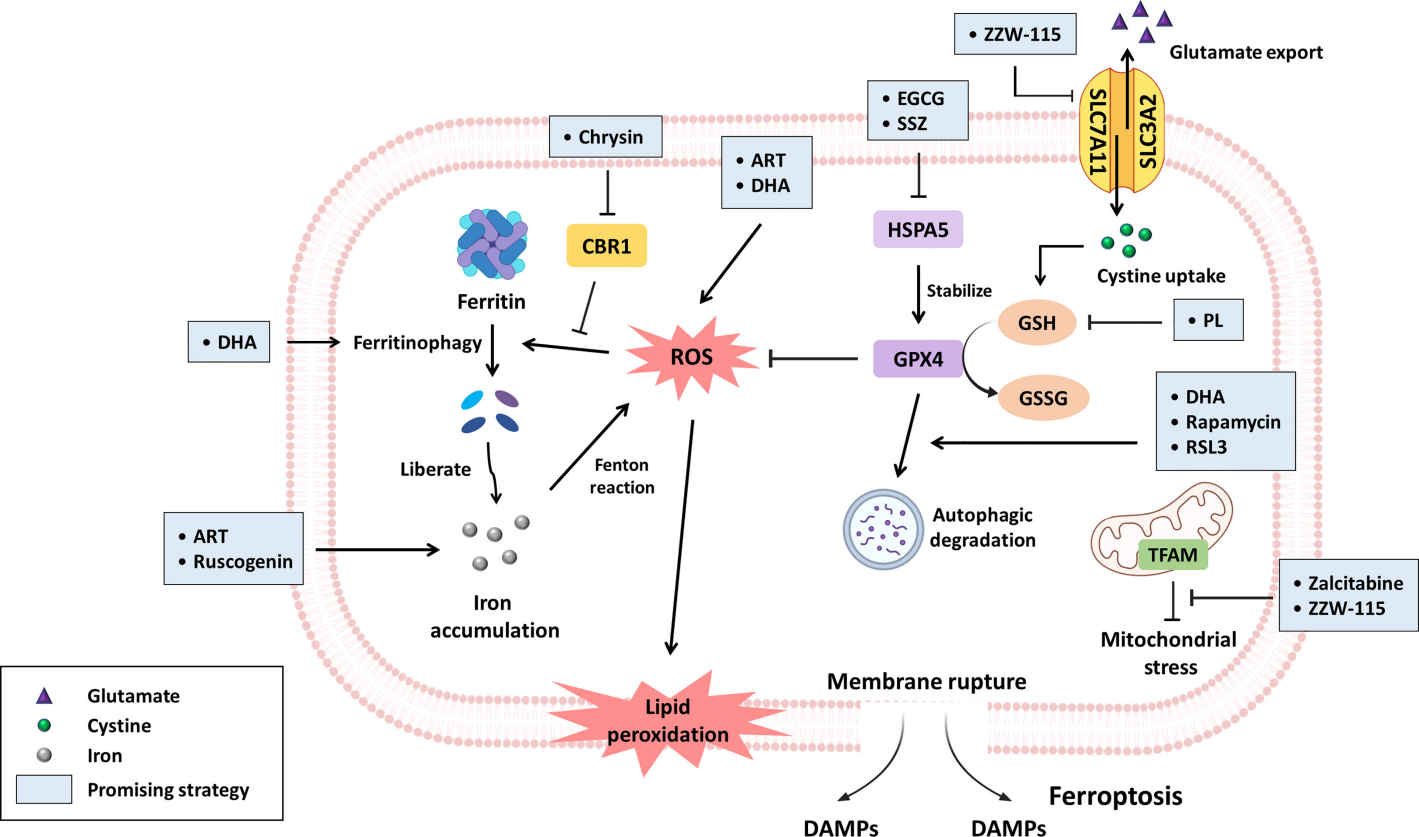
The promising compounds for inhibiting PDAC progression *via* ferroptosis induction. Ferroptosis is primarily initiated through iron accumulation, increased ROS production and lipid peroxidation. With regard to iron accumulation, DHA induces ferritinophagy, and chrysin promotes ROS-mediated ferritinophagy by inhibiting CBR1 expression; in this context, both ART and ruscogenin can contribute to increased intracellular iron levels. Regarding oxidative stress, ART and DHA have been reported to increase ROS generation. GPX4, an important negative modulator of ROS, is stabilized by HSPA5. Nevertheless, EGCG and SSZ have been suggested to block HSPA5, subsequently subverting GPX4 stability. Furthermore, DHA, rapamycin and RSL3 can drive the autophagic degradation of GPX4. GSH synthesized from cystine is a key antioxidant that circumvents ferroptosis, but the product of PL hydrolysis can inhibit GSH generation. SLC7A11 is a subunit of systemic Xc- that facilitates cystine uptake for GSH synthesis, while ZZW-115 downregulates SLC7A11 expression. Zalcitabine and ZZW-115 disrupt TFAM, which is responsible for mtDNA homoeostasis, leading to mitochondrial stress concomitant with lipid peroxidation. Taken together, the aforementioned compounds can induce ferroptosis in PDAC mainly by modulating iron or redox homoeostasis. The promising strategies are highlighted by light blue boxes.

Ferroptosis is characterized by iron accumulation, oxidative stress and lipid peroxidation. Thus, strategies targeting these related modulators might induce ferroptotic cell death. As mentioned above, approximately 90% of PDAC patients harbor *KRAS* mutations; notably, mutant KRAS signaling tends to promote ROS generation (8, 27) mainly through two approaches, including mitochondrial and extramitochondrial ROS production. Liou et al. suggested that mutant KRAS facilitates acinar-ductal metaplasia and progression to pancreatic intraepithelial neoplasias (PanINs), a premalignant stage of PDAC, by inducing mitochondrial ROS (28). In addition, oncogenic KRAS signaling enhances ROS generation *via* the MAPK p38/NADPH oxidase 1 (NOX1) axis (27). Collectively, oxidative stresses play a crucial role in PDAC tumorigenesis as well as progression; however, excessive ROS accumulation eventually triggers cell death instead (29). Hence, PDAC cells might rely on ROS detoxification, to some extent, for survival. A recent study reported that an analysis of the Gene Expression Omnibus (GEO) dataset revealed that key ferroptosis regulators alleviated oxidative stress; these regulators included SLC3A2, SLC7A11 and GPX4, the expression of which were upregulated, especially in GEM-resistant PDAC (30). Furthermore, the finding demonstrated that higher SLC7A11 expression in PDAC is closely linked to poor overall survival (OS) and recurrence-free survival (RFS) (31). According to these findings, PDAC cells are more susceptible to ferroptosis, and therefore, we elucidate several potential strategies (**Figure 1**) for inducing ferroptosis as a PDAC treatment in the following paragraphs.

Artesunate (ART) is an artemisinin derivative that is effective as an antimalarial drug (32). In addition, a recent study suggested that it exerts antitumor activity. Eling et al. demonstrated that ART induces ferroptosis, especially in *KRAS*-mutant PDAC cells, by increasing ROS and intracellular iron levels but does not affect nonmalignant human pancreatic ductal epithelial (HPDE) cells (33).

Human carbonyl reductase 1 (CBR1) is an antioxidant enzyme that protects cells from oxidative stress (34). Furthermore, its role in inducing chemoresistance has been reported (35). ROS accumulation drives autophagic degradation of ferritin and subsequently induces lipid peroxidation as well as ferroptosis. However, CBR1 circumvents ROS-mediated autophagy-dependent ferroptosis, accounting for GEM chemoresistance. Indeed, GEM treatment upregulates CBR1, and its expression has been positively correlated with the clinicopathological characteristics of PDAC. Administration of chrysin (a CBR1 inhibitor) restores ROS accumulation and enhances GEM chemosensitivity *in vitro* and *in vivo* (35).

Cisplatin, a widely used platinum agent, has been evaluated in combination chemotherapy in several ongoing clinical trials (36). Nevertheless, sustained exposure to cisplatin can cause resistance. Du and colleagues discovered that dihydroartemisinin (DHA) can reduce the effective dose of cisplatin and synergistically promote cisplatin-mediated cytotoxicity against PDAC. Mechanistically, this combination induces ferroptosis by enhancing mitochondria-derived ROS production, GPX4 degradation and NCOA4-mediated ferritin degradation. In contrast, administration of the iron chelator deferoxamine (DFO) reverses combination-elicited ferroptosis (37).

As mentioned previously, GPX4 is a pivotal negative regulator of ferroptosis (38). Heat shock 70 kDa protein 5 (HSPA5) not only serves as an endoplasmic reticulum (ER) stress-related chaperone to promote survival but also inhibits ferroptotic cell death (39). Mechanistically, activating transcription factor 4 (ATF4) enhances HSPA5 expression, which promotes the stability of GPX4 and blocks lipid peroxidation. The administration of the HSPA5 inhibitors epigallocatechin gallate (EGCG) or sulfasalazine (SSZ) disrupts the interaction between HSPA5 and GPX4, resulting in ferroptosis and enhancing GEM sensitivity (39).

Piperlongumine (PL), a natural alkaloid derived from *Piper longum* L. in conjunction with cisplatin or paclitaxel has been reported to synergistically induce apoptosis in ovarian cancer (40) and downregulate HER family receptor expression in breast cancer by promoting ROS generation (41). Harshbarger et al. suggested that PL triggers the ferroptotic death of cancer cells but does not affect normal cells, as its hydrolysis product directly binds to GSH and inhibits the antioxidant enzyme glutathione S-transferase Pi 1 (GSTP1), thus contributing to reduced GSH levels with elevated intracellular ROS accumulation (42). Additionally, several combination of agents can be administered to enhance the effects. For example, PL in combination with cotylenin A (CN-A) synergistically induces ferroptosis of MiaPaCa-2 and PANC-1 cells by significantly boosting ROS production (43). SSZ is widely used in inflammatory diseases, such as rheumatoid arthritis and Crohn’s disease (44). The triple combination of PL, CN-A and SSZ significantly inhibits the viability of MiaPaCa-2 and PANC-1 cells but not untransformed mouse embryonic fibroblasts (43).

Rapamycin, also known as sirolimus, is an inhibitor of rapamycin kinase (mTOR) and is widely prescribed in organ transplantation to alleviate rejection (45). A recent study conducted by Liu indicated that rapamycin induces ferroptosis of PDAC cells by autophagy-mediated GPX4 degradation not by suppressing GPX4 mRNA levels. Intriguingly, RSL3, a classic ferroptosis activator, can inhibit mTOR phosphorylation at Ser2448, facilitating autophagy-induced GPX4 degradation. *In vivo*, rapamycin suppresses tumor growth in nude mice subcutaneously implanted with PANC-1 and MiaPaCa2 cells (46).

Ruscogenin, a traditional Chinese medicinal herb, is derived from the root of *Ophiopogon japonicus* (47) and exerts significant anti-inflammatory and antithrombotic effects (48). According to recent research, Ruscogenin can induce ferroptosis of PDAC cells by promoting intracellular iron accumulation concomitant with transferrin upregulation and ferroportin downregulation (47).

Zalcitabine is an antiviral drug for human immunodeficiency virus (HIV)-infected patients (49). Intriguingly, Li and associates suggested that zalcitabine triggers ferroptosis of PDAC cells *via* DNA sensing-mediated autophagy. Stimulator of interferon genes (STING, also known as TMEM173), an ER-related protein, can sense DNA derived from microbial pathogens to induce type I interferon production (50, 51). Zalcitabine promotes mitochondrial DNA stress and ROS levels by degrading mitochondrial transcription factor A (TFAM), which is critical for mitochondrial DNA (mtDNA) homeostasis. This subsequently activates STING/TMEM173 and autophagy-mediated ferroptosis through lipid peroxidation (52).

Nuclear protein 1 (NUPR1), an intrinsically disordered protein lacking a fixed three-dimensional structure, is involved in chromatin remodeling (53, 54). Several studies have reported its role in promoting PDAC tumorigenesis. Hamadi et al. indicated that *NUPR1* protects nutrient-deprived PDAC from stress-mediated cell death. NUPR1 cooperates with KRAS^G12D^ to bypass KRAS^G12D^-induced senescence, which drives PanIN progression (55). Furthermore, NUPR1 also contributes to GEM resistance by upregulating anti-apoptotic properties (56). In contrast, NUPR1 inactivation results in dysfunctional oxidative phosphorylation (OXPHOS) and reduced ATP production, predisposing pancreatic cancer cells to cell death (57). ZZW-115 (an NUPR1 inhibitor) is reported to downregulate TFAM, GPX4 and SLC7A11 expression and increase lipid peroxidation. These effects subsequently trigger the ferroptosis of MiaPaCa-2 cells, including those in xenograft mice (58).

## The Potential of Necroptosis in PDAC Treatment

### Molecular Mechanism of Necroptosis

**Figure 2:**
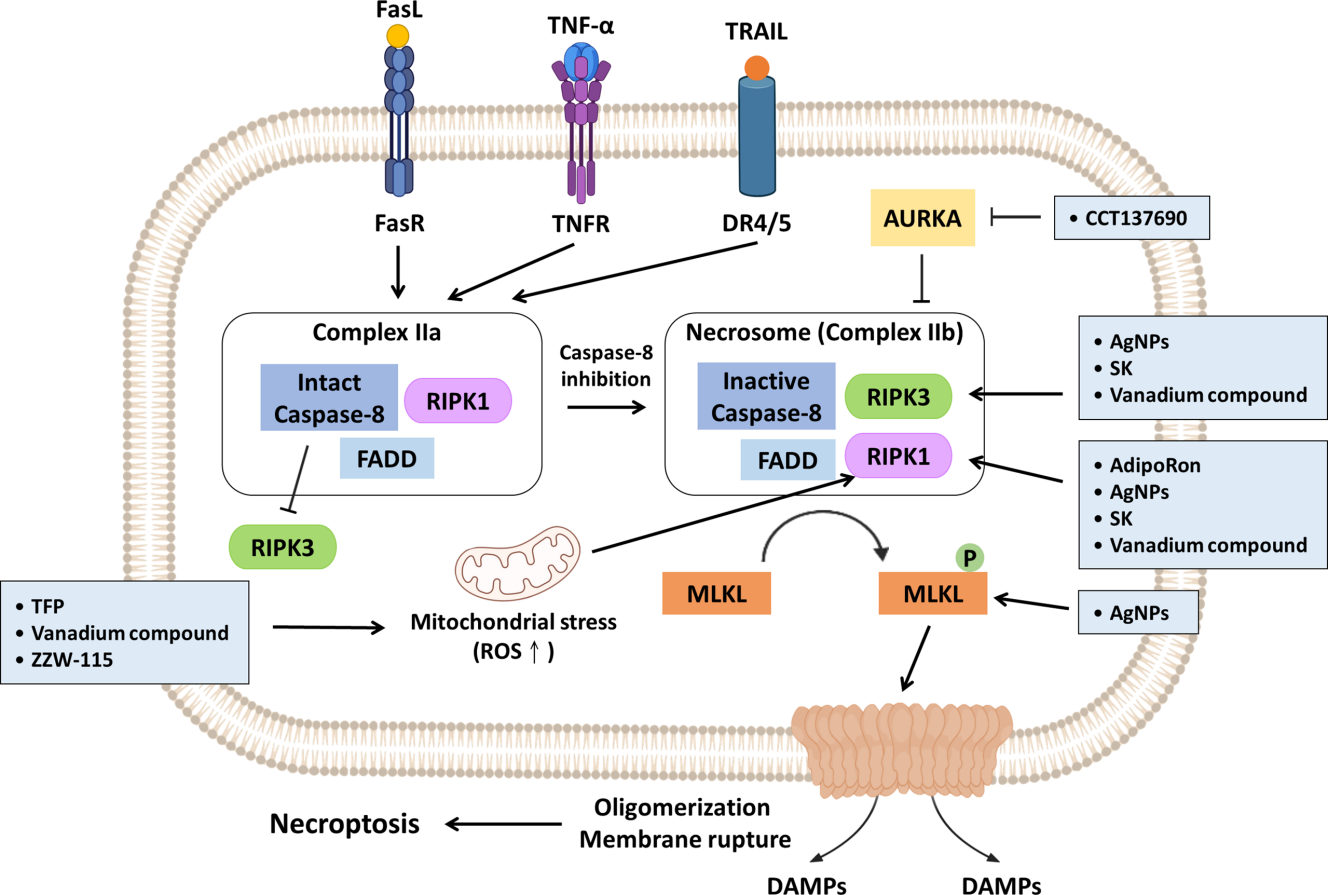
Promising strategies inhibit PDAC progression *via* necroptosis induction. Necroptosis induction is primarily executed by RIPK1, RIPK3 and MLKL. Mitochondrial ROS can facilitate autophosphorylation of RIPK1 and subsequent initiation of necroptosis. TFP, vanadium compound and ZZW-115 have been reported to trigger necroptosis by enhancing mitochondrial stress accompanying increased ROS generation. Aurora kinase A (AUKRA) is a serine/threonine kinase that directly suppresses necrosomes; CCT137690 drives necrosome formation and necroptosis through AUKRA inhibition. AdipoRon, AgNPs, SK and vanadium compounds activate RIPK1; AgNPs, SK and vanadium compounds promote RIPK3 expression. In addition, AgNPs can also directly induce MLKL. The promising strategies are summarized in light blue boxes.

Necroptosis is a form of programmed necrosis and is highly correlated with immunostimulation. It is primarily executed by receptor interacting protein kinase 1 (RIPK1), receptor interacting protein kinase 3 (RIPK3) and mixed lineage kinase like (MLKL). Necroptosis is initiated by death receptors and their corresponding ligands, such as FAS, TNF-α, and TRAIL. After initiation, complex IIa (containing RIPK1, FADD and caspase-8) is formed, and RIPK3 activation is inhibited when caspase-8 remains intact, which triggers apoptosis. Specifically, when caspase-8 activity is inhibited, complex IIb (known as the necrosome), consisting of RIP1, RIP3 and FADD, is formed, which leads to MLKL phosphorylation. Subsequently, phosphorylated MLKL is translocated to the cell membrane where it forms a pore, causing plasma membrane permeabilization (**Table 1**) (23, 24). Furthermore, Zhang et al. discovered a relationship between mitochondrial ROS and necroptosis induction, demonstrating that ROS facilitate the RIPK1 autophosphorylation and RIPK3 recruitment, triggering necroptosis (59). Several potential strategies are discussed in the following paragraphs (**Figure 2**).

### Strategies to Suppress PDAC Growth *via* Necroptosis

Adiponectin (APN) is exclusively secreted by adipocytes and involved in metabolic homoeostasis. A previous study indicated that APN levels are inversely correlated with the development of pancreatic cancer (60). The combination of blood level of APN and interleukin-1 receptor antagonist (IL-1Ra) has been recently used to distinguish PDAC-related diabetes from type II diabetes (61). Akimoto et al. revealed that AdipoRon (an APN receptor agonist) triggers the RIPK1-dependent necroptosis of MiaPaCa-2 cells through mitochondrial dysfunction. *In vivo*, oral administration of AdipoRon suppresses tumor growth and reduces angiogenesis in nude mice subcutaneously injected with MIAPaCa-2 (62).

In addition to their antimicrobial effect, silver nanoparticles (AgNPs) can suppress breast cancer progression (63, 64). Cancer-associated fibroblasts (CAFs), residing in the PDAC stroma, play crucial roles in therapeutic resistance, as they secrete extracellular matrix to form a physical barrier that compromises drug access (65–67). Characterized by a high surface area-to-volume ratio, AgNPs can penetrate dense stroma and exert cytotoxicity in PDAC (68). A recent study revealed that AgNPs decrease the viability of PANC-1 cells in a size- and dose-dependent manner due to necroptosis induction, and this effect was observed with concomitant increases in RIPK1, RIPK3 and MLKL levels (68).

Aurora kinase (AURK) is a serine/threonine kinase and plays a crucial role in mitosis. Hence, targeting AURK seems promising, and several related inhibitors are being evaluated in clinical trials (69). Xie et al. suggested the AURK inhibitor CCT137690 can induce necroptosis of PANC1 PDAC cells *via* the RIPK1/RIPK3/MLKL axis since AURKA is reportedly capable of direct inhibition of necrosome formation. Additionally, the author had established three *in vivo* models to evaluate the antitumor effect of CCT137690. Reduced tumor growth was observed in the three models, and increased CD8+ T cell infiltration was observed in immunocompetent B6 mice implanted with PANC02 cells and mice bearing orthotopic KPC cells. Moreover, higher *AURKA* mRNA expression indicates poor survival of PDAC patients (70).

Shikonin (SK) is a naphthoquinone compound isolated from the root of *Lithospermum erythrorhizon* (71). Chen and associates reported that SK can induce necroptosis by modulating RIPK1 as well as RIPK3 expression. In contrast, silencing RIPK3 expression with a specific RIPK3 short interfering RNA (siRNA) can lead to a reduction in SK-mediated cell death. Administration of SK synergistically enhances GEM efficacy in a PANC-1 xenograft mouse model, reducing the required effective doses of GEM and improving efficacy (72).

Trifluoperazine (TFP) is a widely prescribed antipsychotic drug prescribed for patients with schizophrenia (73). Accumulating evidence has indicated its treatment efficacy on sepsis and its antitumor activity (e.g., in melanoma and colorectal cancer) (73–75). Huang and associates discovered that TFP treatment induces the necroptosis of MiaPaCa-2 cells owing to mitochondrial stress accompanied by reduced ATP level. However, mitochondrial stress is coupled with ER stress to activate the unfolded protein response (UPR), which prevents cells from undergoing programmed cell death. Hence, administration of proteasome inhibitors (e.g., MG-132 or bortezomib) sensitizes PDAC cells to TFP-elicited cytotoxicity (76).

Vanadium compounds have been proposed for the treatment of diabetes mellitus and are effective in inhibiting the proliferation of melanoma through cell cycle arrest and apoptosis mediated by ROS accumulation (77, 78). Kowalski and associates suggested that vanadium compounds, especially those containing organic ligands (e.g., quinolone and phenanthroline), selectively induce both the apoptosis and necroptosis of PANC-1 cells but not nontumorous immortalized pancreas duct epithelial cells (hTERT-HPNE). Mechanistically, vanadium compounds trigger ROS increases and upregulate RIPK1 as well as RIPK3 expression in a dose-dependent manner (79).

As mentioned previously, NUPR1 is a chromatin protein involved in PDAC tumorigenesis. ZZW-115, a derivative of TFP, shows a higher affinity for NUPR1 and induces less neurological toxicity. Mechanistically, it inhibits NUPR1 by restraining nuclear translocation by competing with importins on the nuclear localization signal (NLS) (80). Santofimia-Castaño et al. suggested that inactivation of NUPR1 by ZZW-115 contributes to suboptimal ER stress and subsequent mitochondrial dysregulation. This facilitates increased mitochondrial ROS production concomitant with a dramatic decrease in ATP production, driven by deficient OXPHOS and shifts to glycolysis. Notably, tumor growth is disrupted at low doses of ZZW-115 but reduced at high doses of ZZW-115 in Mia-PaCa-2 cell xenograft mice (57, 81).

## The Potential of Pyroptosis in PDAC Treatment

### Molecular Mechanism of Pyroptosis

**Figure 3:**
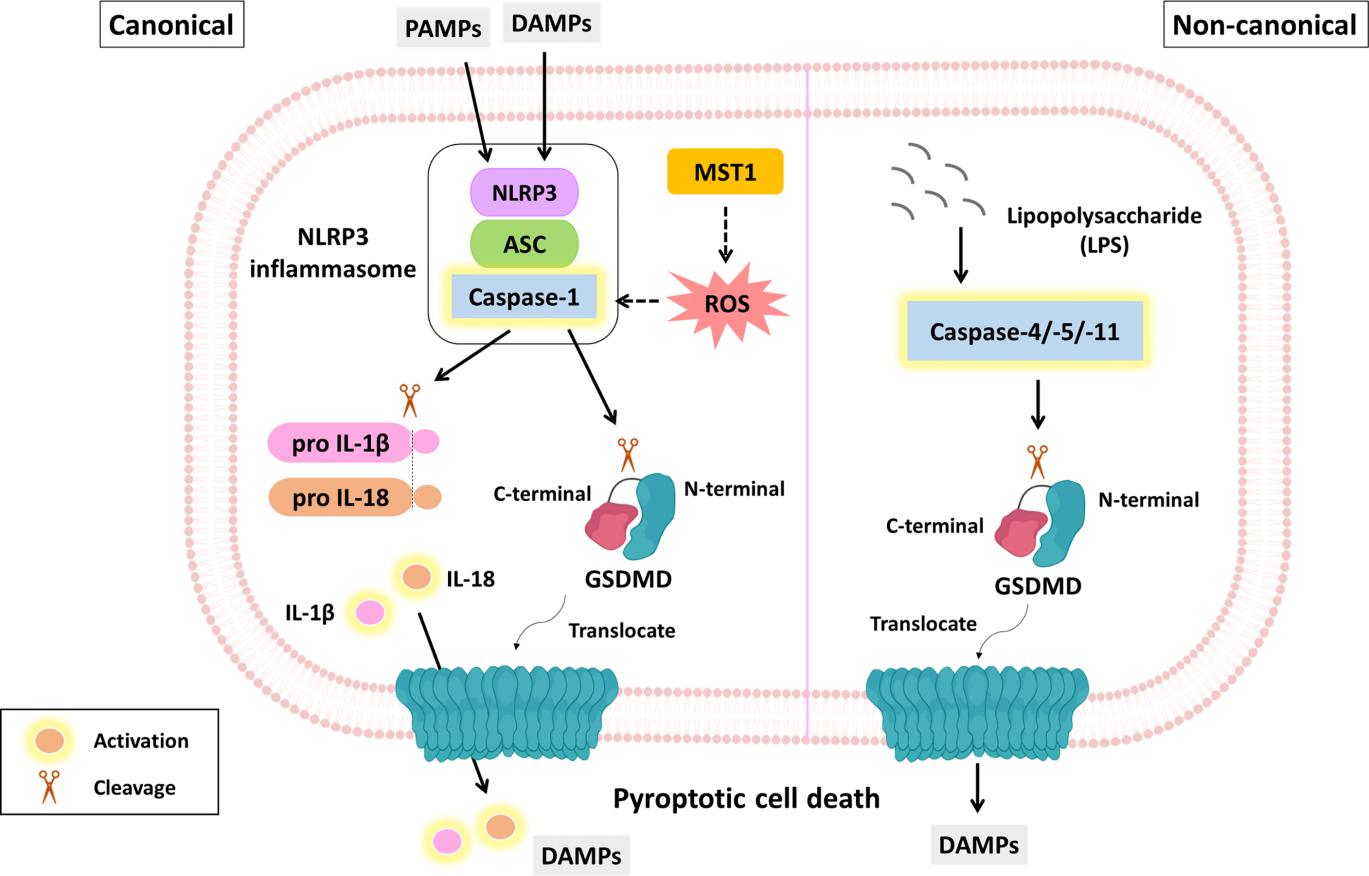
The molecular mechanism of pyroptosis and its role in PDAC suppression. The molecular mechanism of pyroptosis is mainly divided into canonical and noncanonical pathways. The canonical pathway (left panel) is triggered by DAMPs or PAMPs. This process facilitates the formation of inflammasomes, including PRRs (e.g., NLRP3), ASCs and procaspase-1. Activated caspase-1 not only cleaves GSDMD but also contributes to IL-1β and IL-18 maturation. The GSDMD N-terminus is translocated to the plasma membrane where it oligomerizes to form a lytic pore. MST-1 is a component of the Hippo signaling pathway and has been reported to induce caspase-1-dependent pyroptosis *via* ROS. The noncanonical pathway (right panel) is initiated by LPS and executed by caspase-4/-5/-11 not caspase-1, eventually leading to GSDMD cleavage and membrane pore formation. Yellow fluorescence represents active form.

Pyroptosis is also an immunogenic PCD characterized by induced cytoplasmic swelling and plasma membrane rupture caused by the oligomerization of gasdermin (GSDM) protein N-termini. The inflammasome, consisting of pattern recognition receptors (PRRs), apoptosis-associated speck-like protein containing a CARD (ASC) and procaspase-1, is the key mediator of pyroptosis. Pyroptosis is initiated by PRRs (e.g., NLRP3, which is a well-characterized PRR) in the recognition of damage-associated molecular patterns (DAMPs) or pathogen-associated molecular patterns (PAMPs) (25). With regard to molecular mechanisms, pyroptosis is mainly categorized into two pathways: the canonical and noncanonical pathway. Canonical pyroptosis is caspase-1-dependent and contributes to GSDMD cleavage and IL-1β and IL-18 release. Noncanonical pyroptosis is mediated by caspase-4/-5/-11 (caspase-4/-5 in humans and caspase-11 in mice) or caspase-3. The former induces GSDMD cleavage, while the latter facilitates GSDME cleavage (**Table 1**) (**Figure 3**) (26, 82, 83).

### Strategies to Suppress PDAC Growth *via* Pyroptosis

Mammalian STE20-like kinase 1 (MST1), a core component of the Hippo signaling pathway, is a proapoptotic cytoplasmic kinase and is involved in several cellular events, such as proliferation and apoptosis (84, 85). According to clinical specimen analysis, the mRNA level of MST1 is significantly decreased in most PDAC tissues, and its protein level is inversely associated with TNM stage. Cui et al. indicated that MST1 contributes to cell death and suppresses the proliferation and migration of PDAC cells through ROS-dependent pyroptosis independent of the Hippo signaling pathway (**Figure 3**) (86). In contrast, administration of either the caspase-1 inhibitor VX-765 or the ROS scavenger N-acetyl-cysteine (NAC) attenuates MST-1-mediated suppression of PDAC progression (86).

Sonodynamic therapy (SDT) includes specific agents (also known as sonosensitizers), low-intensity ultrasound and molecular oxygen, which noninvasively eliminate tumors through ROS generation (87). 5-Aminolevulinic acid hydrochloride (ALA) is an effective sonosensitizer. Yang and colleagues indicated that ALA-loaded lipid/poly(lactic-co-glycolic acid) (PLGA) microbubble (MB)-mediated SDT induces the pyroptosis of AsPC-1 and BxPC-3 cells because the combination of MBs and ultrasound can facilitate cell membrane permeability and cause cell swelling (88).

## Discussion

**Figure 4:**
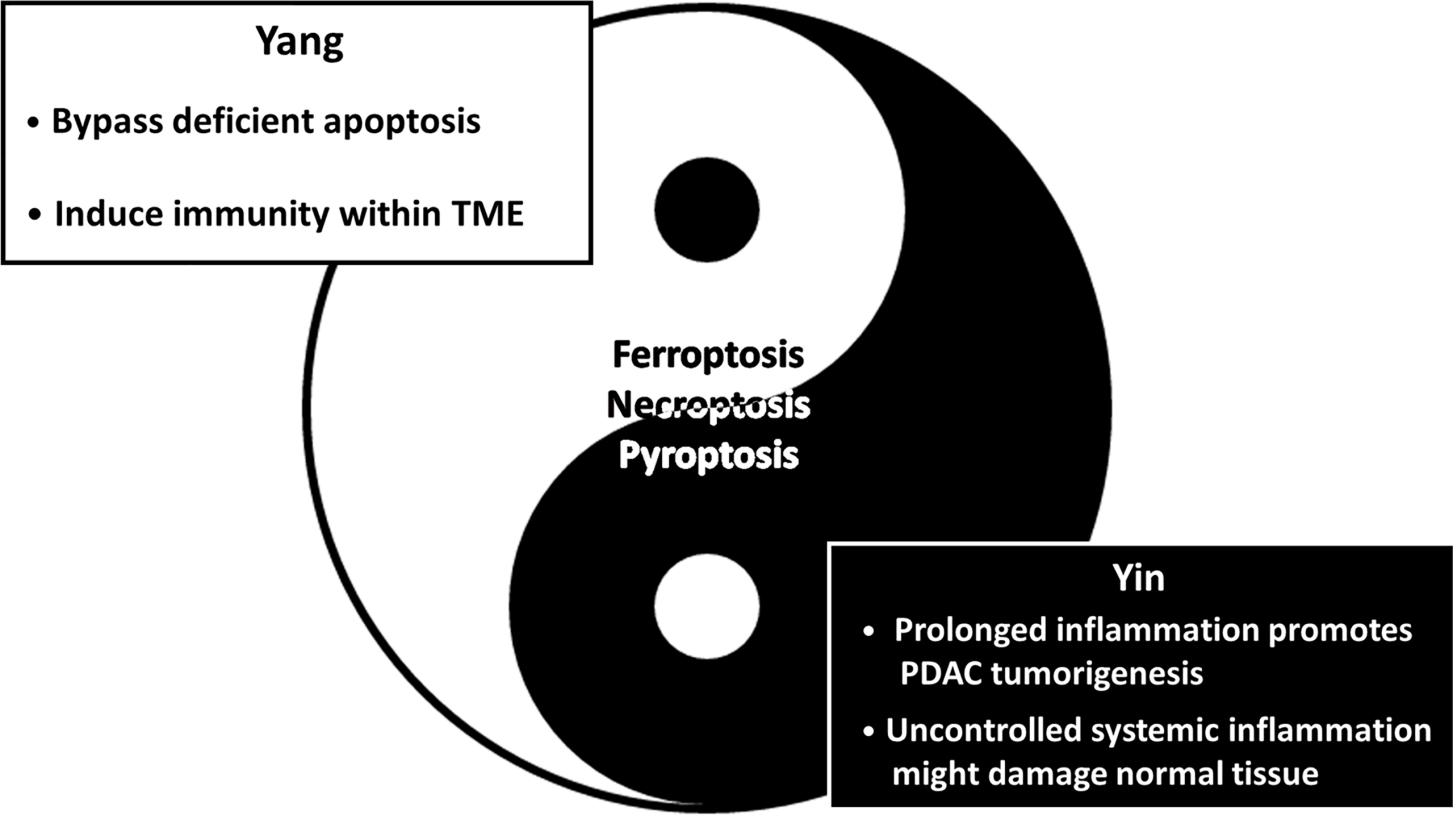
The Yin-Yang theory of inducing nonapoptotic cell death in PDAC suppression. The schematic diagram illustrates the advantages (Yang/Light side) and the disadvantages (Yin/Dark side) of nonapoptotic cell death in PDAC treatments. Simply put, induction of nonapoptotic PCD is promising in PDAC treatment; however, it possibly brings about unwanted clinical outcomes simultaneously. On the one hand, it is capable of eliminating chemoresistant PDAC driven by an aberrant apoptosis mechanism by inducing alternative PCD. Furthermore, ferroptosis, necroptosis and pyroptosis are immunogenic forms of PCD characterized by the release of DAMPs, which probably trigger antitumor immunity. On the other hand, emerging evidence suggests that immunomodulators secreted from immunogenic PCD facilitate immunosuppression. Additionally, uncontrolled systemic inflammation might compromise adjacent normal tissues instead. Collectively, further investigation is urgently needed.

In the past, the molecular mechanism of apoptosis has been extensively investigated, and it has been considered an appropriate drug target to eliminate uncontrollable cell proliferation, achieving considerable success (89). However, a large body of evidence has suggested that several cancer types (e.g., pancreatic cancer, chronic myeloid leukemia and non-small-cell lung cancer) are refractory to chemotherapies or tyrosine kinase inhibitors owing to dysregulated apoptosis (90, 91). Hence, inducing alternative nonapoptotic PCD to eradicate apoptosis-resistant cancer cells has attracted considerable attention in recent years (**Figure 4**) (92). Indeed, it has been estimated that up to 90% of PDAC patients harbor *KRAS* mutations; additionally, *KRAS* and downstream MEK/ERK signaling can induce the degradation of proapoptotic proteins (e.g., Bim) and slow turnover of antiapoptotic proteins (e.g., Mcl-1) (8, 93). Hence, in our review, we elucidate and summarize several potential agents, including compounds and NPs, to suppress PDAC progression by inducing nonapoptotic PCD.

Given that the development and approval of new drugs require substantial investment and time, drug repurposing has gained considerable attention in recent years (94). For instance, Artesunate has been approved by the FDA for the treatment of severe malaria (95); rapamycin is an FDA-approved immunosuppressant administered to organ transplantation patients (96); and Zalcitabine is a nucleoside reverse-transcriptase inhibitor approved by the FDA for HIV treatment (97). Recent studies have demonstrated their potential to induce ferroptosis and exhibit antitumor effects. Hence, drug repositioning seems to be a promising platform through which to explore drugs that induce alternative nonapoptotic PCD in PDAC to overcome antiapoptotic-mediated chemoresistance.

Mitochondria play an important role in the activation of multiple death signaling pathways. The relationship between apoptosis and mitochondria has been well investigated for decades (98). As mentioned previously, ferroptosis is triggered by ROS accumulation and subsequent lipid peroxidation (15); additionally, mitochondria serve as the main site of iron metabolism and cellular ROS generation (99). Mitochondrial ROS are also reported to trigger necroptosis by facilitating RIPK1 autophosphorylation and to induce GSDMD oligomerization, pore formation and subsequent pyroptosis (59, 100). Hence, a nonapoptotic PCD inducer might simultaneously induce several types of cell death, but which type of PCD is predominant needs further study.

Although inducing nonapoptotic PCD seems promising for PDAC treatment, there are several urgent problems that require further investigation. First, because ferroptosis, necroptosis and pyroptosis can lead to plasma membrane rupture and release of cellular contents, they are capable of triggering an immune response within the TME (**Figure 4**). Recent studies have shown that immunogenic PCD might serve as both friend and foe in tumor progression. High mobility group box 1 (HMGB1) is a key DAMP as well as an endogenous danger signal. The three types of nonapoptotic PCD discussed in our review have been suggested to involve HMGB1 release (101–103). Tang et al. revealed that HMGB1 can induce dendritic cell maturation with upregulation of MHC II molecules and promote CD8^+^ T cell infiltration (104). In striking contrast, Das and associates discovered that NLR family pyrin domain containing 3 (NLRP3) inflammasome-derived IL-1β secretion establishes an immunosuppressive TME and results in blocked CD8^+^ T cell infiltration, which drives PDAC tumorigenesis (**Figure 4**) (105). Moreover, recent evidence has suggested that alternatively activated macrophage (M2) polarization can be induced during ferroptotic cell death. Dai et al. suggested that exosomal KRAS^G12D^ released from autophagy-dependent ferroptotic PDAC cells can induce macrophage polarization into the M2 type (106). Another study indicated that 8-hydroxyguanine (8-OHG) released from ferroptotic cells recruits and activates tumor-associated macrophages (TAMs) to secrete IL-6 and nitric oxide synthase 2 (NOS2), promoting tumorigenesis of KRAS-driven PDAC (107). Conditioned medium obtained from necroptotic PDAC cells contains a high level of C-X-C motif chemokine 5 (CXCL5); in addition, C-X-C-motif chemokine receptor-2 (CXCR2) is upregulated in PDAC. These factors induce PDAC migration and invasion *via* the CXCL5/CXCR2 axis (108). Furthermore, upregulation of CXCL5 expression parallels infiltration of immunosuppressive cells (e.g., M2 macrophages and tumor-associated neutrophils), leading to poor prognosis (109). Possible solutions might be combination treatment consisting of nonapoptotic PCD inducers and inhibitors that target immunosuppressive stromal cells to induce synergistic therapeutic effects. For instance, Ando et al. demonstrated that necroptosis-induced migration and invasion were suppressed by a CXCR2 inhibitor (SB225002) (108), and the author suggested that the combination of necroptosis induction therapy and a CXCR2 blocker might suppress its negative impacts. Altogether, nonapoptotic PCD inducers are critical for eliminating antiapoptotic chemoresistant PDAC cells; specific inhibitors disrupt the crosstalk between inflammatory mediators and infiltrated immunosuppressive cells.

As mentioned previously, several nonapoptotic PCD inducers exhibit antitumor effects *in vivo*; xenograft mouse models are chosen as candidates in most studies. Nevertheless, because immunodeficient mice are used as a xenograft models to prevent rejection of xenografts, the role of stromal cells in PDAC progression cannot be elucidated. Thus, the interaction between immunogenic PCD and stromal cells requires further investigation.

The last but not the least problem involving nonapoptotic PCD induction is unwanted inflammatory damage to adjacent or distal normal tissues when the drug target is not sufficiently specific (**Figure 4**). Coating specific antibodies or chemokine receptors on the surface of NPs containing nonapoptotic PCD enhancers may be a solution to promote the trafficking of drugs into the TME. To demonstrate this possibility, Liu and associates modified chimeric antigen receptor-T cells (CAR-T) coated with CXCR2, which more specifically and efficiently targets hepatocellular carcinoma cells with highly expressed CXCR2 ligands (110). A similar strategy might be used to introduce NPs containing nonapoptotic PCD enhancers.

## Author Contributions

S-KH, Y-HC and W-JS wrote the manuscript. K-CC and C-CC conceived of the structure and revised the manuscript. H-HL, W-TC, J-FC, C-YW and C-HY revised the manuscript. All authors read and approved the final manuscript.

## Funding

We thank the following institutions for providing financial support: the Ministry of Science and Technology, Taiwan (grant numbers MOST 109-2314-B-037-069-MY3); NSYSU-KMU joint grants (grant number NSYSUKMU111-P25), and the Kaohsiung Medical University Research Center, Taiwan (grant number KMU-TC109A04); and the Kaohsiung Medical University Hospital (KMUH) (grants KMUH109-9M36 and KMUH110-0M40).

## Conflict of Interest

The authors declare that the research was conducted in the absence of any commercial or financial relationships that could be construed as a potential conflict of interest.

## Publisher’s Note

All claims expressed in this article are solely those of the authors and do not necessarily represent those of their affiliated organizations, or those of the publisher, the editors and the reviewers. Any product that may be evaluated in this article, or claim that may be made by its manufacturer, is not guaranteed or endorsed by the publisher.
